# Combinatorial effects of quercetin and sex-steroids on fluid and electrolytes’ (Na^+^, Cl^-^, HCO_3_^-^) secretory mechanisms in the uterus of ovariectomised female Sprague-Dawley rats

**DOI:** 10.1371/journal.pone.0172765

**Published:** 2017-03-02

**Authors:** Huma Shahzad, Nelli Giribabu, Kamarulzaman Karim, Normadiah M. Kassim, Sekaran Muniandy, Naguib Salleh

**Affiliations:** 1 Department of Physiology, Faculty of Medicine, University of Malaya, Lembah Pantai, Kuala Lumpur, Malaysia; 2 Department of Anatomy, Faculty of Medicine, University of Malaya, Lembah Pantai, Kuala Lumpur, Malaysia; 3 Department of Biochemistry, Faculty of Medicine and Biomedical Sciences, MAHSA University, Jalan Elmu, Off Jalan University, Kuala Lumpur, Malaysia; Max Delbruck Centrum fur Molekulare Medizin Berlin Buch, GERMANY

## Abstract

Dysregulation of uterine fluid environment could impair successful reproduction and this could be due to the effect of environmental estrogens. Therefore, in this study, effect of quercetin, an environmental estrogen on uterine fluid and electrolytes concentrations were investigated under sex-steroid influence. Ovariectomised adult female Sprague-Dawley rats were given 10, 50 or 100mg/kg/day quercetin subcutaneously with 17-β estradiol (E) for seven days or three days E, then three days E plus progesterone (P) (E+P) treatment. Uterine fluid secretion rate, Na^+^, Cl^-^ and HCO_3_^-^ concentrations were determined by *in-vivo* perfusion. Following sacrifice, uteri were harvested and levels of the proteins of interest were identified by Western blotting and Realtime PCR. Distribution of these proteins in the uterus was observed by immunofluorescence. Levels of uterine cAMP were measured by enzyme-linked immunoassay (EIA). Administration of quercetin at increasing doses increased uterine fluid secretion rate, Na^+^, Cl^-^ and HCO_3_^-^ concentrations, but to the levels lesser than that of E. In concordant, levels of CFTR, SLC4A4, ENaC (α, β and γ), Na^+^/K^+^-ATPase, GPα/β, AC and cAMP in the uterus increased following increased in the doses of quercetin. Co-administration of quercetin with E caused uterine fluid secretion rate, Na^+^, Cl^-^ and HCO_3_^-^ concentrations to decrease. In concordant, uterine CFTR, SLC26A6, SLC4A4, ENaC (α, β and γ), Na^+^/K^+^-ATPase, GPα/β, AC and cAMP decreased. Greatest effects were observed following co-administration of 10mg/kg/day quercetin with E. Co-administration of quercetin with E+P caused uterine fluid Na^+^ and HCO_3_^-^ concentrations to increase but no changes in fluid secretion rate and Cl^-^ concentration were observed. Co-administration of high dose quercetin (100 mg/kg/day) with E+P caused uterine CFTR, SLC26A6, AC, GPα/β and ENaC (α, β and γ) to increase. Quercetin-induced changes in the uterine fluid secretion rate and electrolytes concentrations could potentially affect the uterine reproductive functions under female sex-steroid influence.

## Introduction

Precise regulation of uterine fluid volume and electrolytes concentrations by female sex hormones, namely estrogen and progesterone is essential for successful reproduction [1, 2]. In mice, excessive uterine fluid accumulation as observed in hyperestrogenic state could lead to aberrant embryo implantation (3). In humans, disturbances in uterine fluid volume could lead to infertility [3]. Infertile women have been found to have higher concentration of glycodelin, an endometrial protein than the fertile women at luteinizing hormone day-1 (LH+1) which could be due to the changes in uterine fluid volume [4]. Excessive accumulation of fluid in the uterine cavity could also interfere with endometrial receptivity development that is needed for successful embryo implantation [5].

Besides the abnormal changes in the amount of fluid in uterus, abnormal changes in uterine fluid Na^+^, Cl^-^ and HCO_3_^-^ concentrations could also interfere with embryo implantation and other uterine reproductive processes. Electrolytes such as HCO_3_^-^ is essential for sperm transport and capacitation [6]. In mice, aberrant expression of Cl^-^ and HCO_3_^-^ transporters in the endometrium could impair embryo implantation [7]. Factors that interfere with the regulation of uterine fluid electrolytes under sex-steroid influence include exposure to phytoestrogen genistein [8] and high dose testosterone [9]. In addition, pathological condition such as hydrosalpinx, associated with excessive fluid accumulation in the Fallopian tube [5] could also to interfere with uterine reproductive function.

Female sex hormones namely estrogen and progesterone are known to regulate the volume and electrolytes compositions of uterine fluid via affecting membrane transporters’ expression in the uterus such as Cystic Fibrosis Transmembrane Regulator (CFTR), a cAMP-activated Cl^-^ and HCO_3_^-^ channels [8]. In addition, expression of Cl^-^/HCO_3_^-^ exchanger (SLC26A6) that transport HCO_3_^-^ into the uterine lumen in exchange with Cl^-^ and Na^+^/HCO_3_^-^ co-transporter, that transport both Na^+^ and HCO_3_^-^ into the uterine lumen could also be affected by female sex hormones [2, 10]. Expression of epithelial sodium channel (ENaC) and Na^+^/K^+^-ATPase which are involved in Na^+^ reabsorption from the uterine lumen were also found to be regulated by female sex hormones [11]. Under the influence of estrogen, CFTR, SLC26A6 and SLC4A4 expressions in uterus increased [2], whilst under the influence of progesterone, ENaC α, β and γ and Na^+^/K^+^-ATPase expression in uterus were increased [11].

Environmental estrogens have been reported to interfere with the regulation of uterine fluid environment. Genistein for example, could affect uterine fluid volume and expression of membrane transporters in the endometrium [12]. In view that quercetin is a phytoestrogen [13], therefore there is a possibility that this compound could affect the volume and electrolytes concentration of the uterine fluid. Quercetin is a phenolic compound widely available in fruits and vegetables such as onions, broccolis, apples and grapes [14]. Nowadays, quercetin is used as a dietary health supplement [15], with multiple claim benefits in protecting against osteoporosis, cancer, pulmonary and cardiovascular diseases and ageing [15]. This compound was reported to act on estrogen-responsive tissues such as uterus [16]. Amongst the documented uterine effects of quercetin include inducing morphological [17] proliferative changes [18]. Quercetin has also been reported to regulate uterine inflammatory events [19] and was able to interfere with uterine smooth muscles contraction [20]. Therefore, we hypothesized that quercetin could affect the uterine fluid environment i.e. volume and electrolytes concentration in the presence of female sex hormones. The aims of this study were to investigate changes in uterine fluid secretion volume and electrolytes (Na^+^, Cl^-^, HCO_3_^-^) concentrations under quercetin influence in the presence of female sex-steroids and the uterine mechanisms that are likely involved in mediating these effects.

## Materials and methods

### Animal preparation

Three month-old adult female Sprague-Dawley (SD) rats, weighing 225 ± 10g were housed in a clean and well ventilated environment with standardized conditions (lights 12 h from 06:00 h to 18:00 h: room temperature 25 ± 2°C; with 5–6 animals per cage). Rats were given free access to rodent pellet (Harlan Diet, Rossdoff, Germany) and tap water *ad libitum*. Ethical approval was granted by Institutional Animal Care and Use Committee, University of Malaya with ethics number 2013-07-15/FIS/R/NS. Quercetin, E, P and peanut oil were purchased from Sigma Aldrich Co (St. Louis, MO, USA).

Twenty one days prior to sex-steroids and quercetin treatment, bilateral ovariectomy was performed under isoflurane anesthesia to eliminate the variations in the sex-steroid levels. Ovariectomy was performed according to the methods as previously described [1]. Quercetin was dissolved in 30% DMSO and was serially diluted to achieve the desired final concentrations prior to mixing with peanut oil. Final quercetin concentrations achieved were as follows: 10mg/kg/day quercetin (2mg/0.1ml peanut oil/day), 50mg/kg/day quercetin (10mg/0.1ml peanut oil/day) and 100mg/kg/day quercetin (20mg/0.1ml peanut oil/day). Drugs were injected behind the neck scruff for seven (7) consecutive days. Animals were then divided into the following groups, with N = 6 per group:

***Study design 1*: Quercetin injection only**
*Group 1*: 7 days treatment with peanut oil (vehicle)—negative control (C)
*Group 2*: 7 days treatment with 10 mg/kg/day quercetin (10G)
*Group 3*: 7 days treatment with 50 mg/kg/day quercetin (50G)
*Group 4*: 7 days treatment with 100 mg/kg/day quercetin (100G)
*Group 5*: 7 days treatment with 0.8 x10^-^4 mg/kg/day E

***Study design 2*: Quercetin plus female sex-steroids**
*Group 1*: 7 days treatment with peanut oil (vehicle); C
*Group 2*: 7 days treatment with 17-β estradiol (1 μg/kg/day); E
*Group 3*: 7 days treatment with 17-β estradiol (1 μg/kg/day) plus quercetin at 10 mg/kg/day; E+10Q
*Group 4*: 7 days treatment with 17-β estradiol (1 μg/kg/day) plus quercetin at 50 mg/kg/day; E+50Q
*Group 5*: 7 days treatment with 17-β estradiol (1 μg/kg/day) plus quercetin at 100 mg/kg/day; E+100Q
*Group 6*: 3 days 17-β estradiol (1 μg/kg/day), a day no treatment, then 3 days progesterone (20 mg/kg/day); E+P
*Group 7*: 3 days 17-β estradiol (1 μg/kg/day), a day no treatment, then 3 days progesterone (20 mg/kg/day) with quercetin 10 mg/kg /day from day 1 till day 7; E+P10Q
*Group 8*: 3 days 17-β estradiol (1 μg/kg/day), a day no treatment, then 3 days progesterone (20 mg/kg/day) plus quercetin 50 mg/kg /day from day 1 till day 7; E+P50Q
*Group 9*: 3 days 17-β estradiol (1 μg/kg/day), a day no treatment, then 3 days progesterone (20 mg/kg/day) plus quercetin 100 mg/kg /day from day 1 till day 7; E+P100Q

Doses of quercetin were chosen according to the previously reported doses [17, 21, 22]. Drugs were administered subcutaneously to avoid the first-pass effect (hepatic metabolism) [23]. E was given to mimic changes in hormonal profile in the first half of the reproductive cycle while E followed by E+P were given to mimic changes in the hormonal profile in first half followed by the second halves of the female reproductive cycle. A day after last injection, rats were anesthetised by administering 80mg/kg b.w ketamine and 8mg/kg b.w xylazine intramuscularly, prior to *in-vivo* uterine perfusion. At the end of the perfusion experiment, anesthetized rats were sacrificed by cervical dislocation and uteri were immediately harvested for protein and mRNA expression analyses and analysis of protein distribution.

### *In-vivo* uterine perfusion

*In-vivo* uterine perfusion was performed to identify changes in uterine fluid secretion rate, according to the previously described method [2]. In brief, anesthetized rats were placed on a heat pad to maintain their constant body temperature and incision was made at both flanks to insert an in-going tube pre-filled with perfusate from distal end of the uterine horn. Out-going tube was inserted and tied *in-situ* at the uterocervical junction. A syringe-driven infusion pump (Harvard Apparatus, USA) was used to deliver the perfusate into the lumen at a constant rate of 0.75μl/min. The in-going tube, uterine horn and out-going tube were placed at the same level to minimize the gravitational effect.

The perfused fluid was collected over a period of 3 h into small, pre-weighed polythene tubes with covered top to minimize evaporation. Perfusate contains the following electrolytes’ compositions: 110.0mmol/l NaCl, 14.3mmol/l Na_2_HCO_3_, 1.0 mmol/l Na_2_HPO_4_, 15 mmol/l KCl, 0.8 mmol/L MgSO4, 10.0 HEPES, 1.8 mmol/l CaCl_2_, 5.5 mmol/l glucose and pH 7.34, which were chosen to closely mimic the normal uterine fluid composition [1]. To determine the uterine fluid secretion rate, net weight of the collected fluid was divided by total perfusion time (180 min). Tube weight was measured by using an electronic balance (EL3002 METTLER TOLEDO) prior to and after perfusate collection. Uterine fluid secretion rate was calculated from differences between tube weight prior to and after perfusate collection.

HCO_3_^-^ concentration in the collected perfusate was determined by using enzymatic assay. In this assay, phosphoenolpyruvate carboxylase (PEPC) and malate dehydrogenase (MDH) were used. The end products of the enzymatic reactions were measured using a spectrophotometer at absorbance wavelengths of 405 or 415 nm. Changes in colour were directly proportional to HCO_3_^-^ concentration in the samples. Na^+^ and Cl^-^ concentrations were measured by direct method using Ion Selective Electrode (ISE), which is voltage-dependent on ions levels in the solution.

### Quantification of mRNA expression levels by Realtime PCR (qPCR)

Whole uterine tissues were kept in RNALater solution (Ambion, Austin, TX, USA), prior to RNA extraction. Total RNA was freshly isolated from rat uteri by using RNeasy Plus Mini Kit (Qiagen, Valencia, CA, USA). RNA purity and concentration of were assessed by 260/280 UV absorption ratios (Gene Quant 1300, GE Healthcare UK Limited, Buckinghamshire, UK). Two steps Real-time PCR was used to calculate the gene expression with application of TaqMan^®^ RNA-to-CT 1-Step Kit (Ambion, Austin, TX, USA). This kitis highly sensitive [24]. Reverse transcription into cDNA was performed by using high capacity RNA-to-cDNA Kit (Applied Biosystems, Foster City, CA, USA). Controls include amplification performed on the samples identically prepared with no reverse transcriptase (-RT) as well as amplifications performed with no added substrate.

The assay used, TaqMan^®^- ID number Rn01455979_m1, Rn01445892_m1, Rn00580652_m1, Rn00561892_ml and Rn00566891_m1) (Applied Biosystem, USA) with specific RNA sequence amplified 73bp segment *Cftr*, 67bp segment *Slc26a6*, 114bp segment *α-ENaC*, 84bp segment *β-ENaC* and 76bp segment *γ-ENaC*. Target assay was validated *in-silico* by using whole rat genome and *in-vitro* by using whole rat cDNA. These ensured that target sequences were detected (Applied Biosystems, Foster City, CA, USA). *Gapdh* and *Hprt* were used as reference genes as they were stably expressed in the uterus [25].

PCR program included 2 min at 50°C for UNG activity, 20 sec 95°C activation of ampliTaq gold DNA polymerase and 1 min denaturation at 95°C, 20 sec and annealing/ extension at 60°C for 1 min. Denaturing and annealing were performed for 40 cycles. All measurements were normalized by using GenEx software (MultiD, Goteburg, Sweden), followed by Data Assist v3 software from Applied Biosystems, MA, USA. These softwares calculated the RNA fold changes. All experiments were performedt in triplicates. Data were analyzed according to comparative Ct (2^-ΔΔCt^) method.

### Visualization of protein distribution by immunofluorescence

Uterine tissues were fixed overnight in 4% paraformaldehyde (PFD), prior to processing. Tissue processing include dehydration through increasing concentrations of ethanol, where the tissues were then cleared in chloroform and blocked in paraffin wax. Following these, tissues were cut into 5μm sections, deparaffinized in xylene and rehydrated in reducing concentrations of ethanol. Tri sodium citrate (pH 6.0) was used for antigen retrieval. Tissues were blocked in appropriate 10% normal serum (Santa Cruz Biotechnology, Santa Cruz, CA, USA) for 1 h at room temperature prior to incubation with CFTR, SLC26A6, SLC4A4-B, α-ENaC, β-ENaC, *γ*-ENaC and Na^+^/K^+^-ATPase primary antibodies (details shown in Table 1) at a dilution of 1:100 in PBS in appropriate serum. After three times rinsing with PBS, sections were incubated with donkey anti-goat IgG-FITC for CFTR and SLC26A6 (details in Table 1) at a dilution of 1:250 in PBS with 1.5% normal blocking serum at room temperature for 45 min. The slides were rinsed three times with PBS and were mounted with Ultracruz mounting medium (Santa Cruz Biotechnology, Santa Cruz, CA, USA), counterstained with DAPI to visualize the nuclei. All images were viewed under Nikon Eclipse 80i camera attached to the light microscope.

**Table 1:** List of primary and sencondary antibodies used in the present study.

Primary antibody	Serum	Secondary antibody
CFTR (sc-8909; goat polyclonal IgG)	Donkey sc-2044	Donkey anti-goat–FITC sc-2024
SLC26A6 (sc-26728; goat polyclonal IgG)	Donkey sc-2044	Donkey anti-goat–FITCsc-2024
SLC24A4 (sc-162215; rabbit polyclonal IgG)	Donkey sc-2044	Goat anti rabbit–FITC sc-2012
αENaC (sc-22237; goat polyclonal IgG)	Donkey sc-2044	Donkey anti-goat- FITC sc-2024
β- ENaC (sc-25354; mouse monoclonal IgG)	Goat sc-2043	Goat anti-mouse- FITC sc-2010
γ- ENaC (sc-21014; rabbit polyclonal IgG)	Goat sc-2043	Goat anti rabbit–FITC sc-2012
Na^+^/K^+^ ATPase α (sc-28800; rabbit polyclonal IgG)	Goat sc-2043	Goat anti-rabbit- FITC sc-2012

### Uterine cAMP measurement

Freshly isolated uteri were homogenized in 5 volume (ml/g tissue) ice-cold 5% trichloroacetic acid (TCA) by using Polytron-type homogenizer. Homogenate was centrifuged at 1,500×g for 10 min. Supernatant was transferred into test tubes, extracted three times using a water-saturated ether. Ether fraction was then discarded and residual ether in the aqueous fraction was removed by heating at 70°C on hot plate, 5 min. Levels of cAMP in homogenates were measured by using enzyme immunoassay (EIA) kit (Cayman Chemical Company, Ann Arbor, MI, USA; Item No. 581001), according to the manufacturer’s guideline. Absorbance was read at 405nm in a micro-plate reader (Hidex, Turku; Finland) and the units were expressed in pmol/ml.

### Protein quantifications by Western blotting

The uterine horns were snap frozen in liquid nitrogen and then stored at −80°C prior to protein extraction in PRO-PREP solution (Intron, Korea). 80μg proteins were mixed with loading dye, separated in SDS-PAGE and transferred onto PVDF membrane (BIORAD, USA). The membranes were blocked with 5% BSA for 90 min at room temperature and were separately exposed to CFTR, SLC26A6, SLC4A4-B, α-ENaC, β-ENaC and γ-ENaC, AC, Na^+^/K^+^-ATPase, GPα and GPβ primary antibodies (details in Table 1) at concentration of 0.2μg/ml (1:1000) in PBS containing 1% BSA and tween-20 overnight. GAPDH and β-actin at concentration of 0.2μg/ml (1: 500) was used as loading control. All antibodies were purchased from Santa Cruz Biotechnology, CA, USA.

Blots were rinsed three times in PBS-T, 5 min each. Membranes were then incubated with anti-rabbit and anti-goat horseradish peroxidase (HRP) conjugated secondary antibody (details in Table 1) at a concentration of 0.2μg/ml (1:2000), for 1 h and protein bands were visualize by using Opti-4CN^™^ Substrate Kit (Bio-Rad, USA). Images of the bands were captured by using a gel documentation system. Density of the bands was quantified by using Image J software (NIH version 1.46j; National Institutes of Health, Bethesda, MD, USA). Ratios of each target protein/GAPDH or β-actin was determined and these were considered as the expression levels of the targets.

### Statistical analysis

Statistical differences were evaluated by using Student's t-test and one-way analysis of variance (ANOVA) (GraphPad Prism@ software). p<0.05 was considered as significant. Tukey p*ost-hoc* statistical power analysis was performed and all values were >0.8 which indicated adequate sample size.

## Results

### Effects of quercetin-only and quercetin plus sex-steroids on uterine fluid secretion rate and electrolytes (Na^+^, Cl^-^ and HCO_3_^-^) concentrations

**Fig 1:**
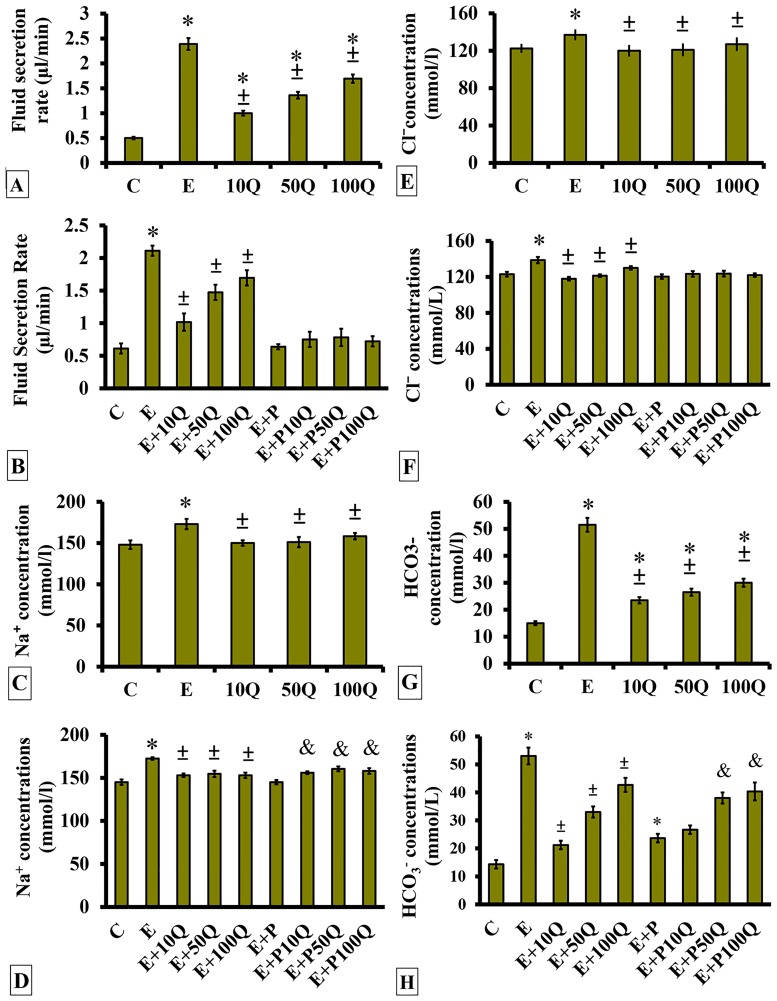
Changes of uterine fluids (A&B) secretion rate, (C&D) Na^+^ concentration, (E&F) Cl^-^ concentration and (G&H) HCO_3_^-^ concentration in non sex-steroid and sex-steroid replaced ovariectomized rats following quercetin treatment. Data were expressed as mean +/- SEM. N = 6 per treatment group. *p < 0.05 compared to C, ±p<0.05 compared to E. ^&^ p<0.05 compared to E+P.

In ovariectomised, non sex-steroid-treated rats, uterine fluid secretion rate was highest following E treatment (Fig 1A). Treatment with quercetin resulted in a dose-dependent increase in uterine fluid secretion rate. However, maximal rate observed following highest dose quercetin treatment did not surpass the levels following E treatment. In ovariectomized rats which received E treatment, uterine fluid secretion rate markedly reduced following concomitant quercetin administration (Fig 1B). Co-administration of quercetin doses at 50 and 100 mg/kg/day to E-treated rats resulted in reduction of the uterine fluid secretion rate, but to the levels significantly lesser than following co-administration of 10mg/kg/day quercetin with E. Meanwhile, in rats receiving E+P treatment, uterine fluid secretion rate was lower when compared to rats receiving E treatment and this was not affected by quercetin co-administration.

In ovariectomised, non-sex-steroid-treated rats, uterine fluid Na^+^ concentration was highest following E treatment (Fig 1C). Treatment with quercetin did not causes significant changes in uterine fluid Na^+^ concentration, but the levels were lower than in rats receiving E treatment. In E-treated ovariectomised rats, co-administration of 10, 50 and 100mg/kg/day quercetin resulted in a significant decrease in uterine fluid Na^+^ concentration (p<0.05) (Fig 1D). Uterine fluid Na^+^ concentration in E+P-treated rats was lower than in E-treated rats (p<0.05) and was slight but significantly increased following quercetin co-administration.

Changes in uterine fluid Cl^-^ concentration followed changes in Na^+^ concentration. In ovariectomised, non-sex-steroid- treated ovariectomized rats, administration of quercetin did not cause significant changes to uterine fluid Cl^-^ concentration, with the levels did not surpass that of E (Fig 1E). In ovariectomised sex-steroid replaced rats, E treatment resulted in highest uterine fluid Cl^-^ concentration which was markedly decreased following quercetin treatment (Fig 1F). Lesser decrease was observed following co-administration of high dose as compared to low dose quercetin. In E+P treated rats, uterine fluid Cl^-^ concentration was significantly lower than in E-treated rats, and this was not affected by concomitant quercetin treatment.

In non-sex-steroid-treated ovariectomised rats, uterine fluid HCO_3_^-^ concentration was the highest following E treatment, approximately 5-times higher than control (Fig 1G). Increased in uterine fluid HCO_3_^-^ concentration was observed following increasing doses of quercetin, but the levels did not surpass that of E. The highest HCO_3_^-^ concentration which was observed in E-treated ovariectomised rats markedly decreased following co-administration of low dose (10 mg/kg/day) quercetin by approximately 2 ½ fold (Fig 1H). Lesser decrease was observed at higher quercetin doses. Meanwhile, in E+P treated rats, uterine fluid HCO_3_^-^ concentration, which was lower than that observed following E treatment, was markedly increased in rats receiving 50 and 100 mg/kg/day but not 10 mg/kg/day quercetin treatment.

### Effects of quercetin-only and quercetin plus sex-steroids on Adenylate Cyclase II (AC II), G-protein α and β and cAMP levels in the uterus

**Fig 2:**
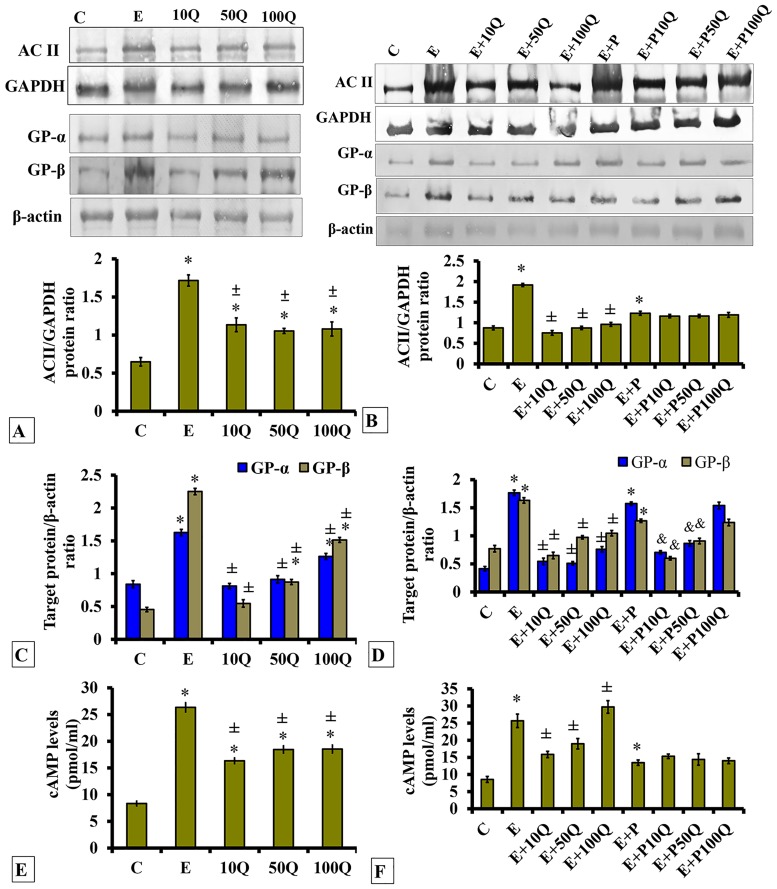
Representative Western blot image of AC, GPα and GPβ, (A&B) analyses of band intensity for AC/GAPDH (C&D) analyses of band intensity for GPα or GPβ/β-actin and (E&F) levels of cAMP in ovariectomized, non-sex-steroid treated and sex-steroid treated rats receiving concomitant treatment with quercetin. Data were expressed as mean +/- SEM. N = 6 per treatment group. *p < 0.05 compared to C, ±p<0.05 compared to E. ^&^ p<0.05 compared to E+P.

AC II levels in uterus of ovariectomised rats markedly increased following E treatment (Fig 2A). A slight increase in AC II levels was observed following increasing doses of quercetin treatment, however the levels did not surpass that in E-treated rats. In ovariectomised, sex-steroid replaced rats, highest AC II levels observed following E treatment and these were markedly reduced following co-administration of quercetin (Fig 2B). Greatest reduction was observed following co-administration of 10mg/kg/day quercetin with lesser reduction at higher quercetin doses (50 and 100mg/kg/day). Meanwhile, in E+P treated ovariectomised rats, AC II levels were lower than in E-treated rats, and these were not affected by quercetin co-treatment.

Levels of GPα and GPβ in uterus were highest in E-treated ovariectomised rats (Fig 2C). GPα and GPβ levels increased with increased in quercetin doses, but to the levels that did not surpassed that of E. The highest GPα and GPβ levels were observed in ovariectomised rats receiving E treatment (Fig 2D), and these were markedly decreased following co-administration of quercetin. Co-administration of highest quercetin dose (100mg/kg/day) with E caused lesser decrease in GPα and GPβ levels as compared to administration of lower dose quercetin. Meanwhile, in E+P-treated rats, GPα and GPβ levels, which were lower than in E-treated rats, decreased following concomitant quercetin treatment with the highest decrease was observed at low quercetin dose.

Levels of cAMP in uterus were highest in rats which received E-only treatment (Fig 2E). Following administration of quercetin, the levels were lower than in E-treated rats but were higher than control. Meanwhile, in sex-steroid replaced ovariectomised rats, the highest cAMP levels which were observed following E treatment was markedly reduced following concomitant administration of 10mg/kg/day and 50 mg/kg/day quercetin (p<0.05) (Fig 2F). However, following co-administration of 100 mg/kg/day quercetin with E, no differences in cAMP levels was observed. Meanwhile, in E+P-treated rats, cAMP levels were approximately two-times lower than in E-treated rats, and these were not affected by concomitant quercetin treatment.

### Effects of quercetin-only and quercetin plus sex-steroids on CFTR protein and mRNA expression and its protein distribution in the uterus

**Fig 3:**
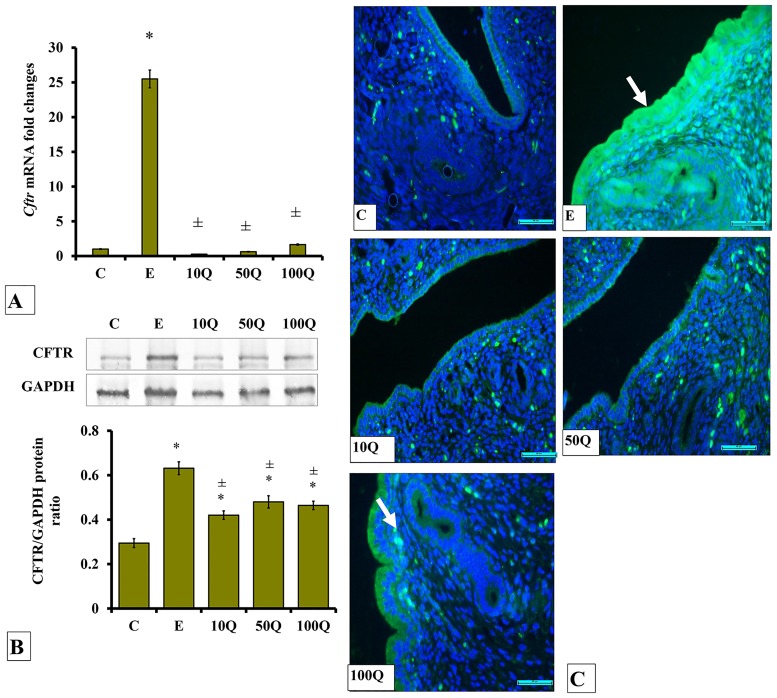
(A) *Cftr* mRNA levels, (B) Representative Western blot image and ratio of CFTR /GAPDH and (C) CFTR distribution in the endometrium of ovariectomized, quercetin-treated rats. Data were expressed as mean +/- SEM. N = 6 per treatment group. For A-C, *p < 0.05 compared to C, ±p<0.05 compared to E. ^&^ p<0.05 compared to E+P.

In ovariectomised, non-sex-steroid-treated rats, *Cftr* mRNA levels markedly increased following E-only treatment (25-times higher than control) (Fig 3A). Treatment with quercetin did not cause marked changes to the *Cftr* mRNA level. CFTR protein level in uterus of ovariectomised, non-sex-steroid-treated rats was highest following E treatment (Fig 3B). Quercetin treatment caused lower CFTR protein level in uterus as compared to E. Increasing quercetin doses caused increased CFTR protein level, however did not surpass that of E-only treatment. Immunofluorescence images (Fig 3C) show CFTR protein was highly distributed at the apical membrane of endometrial luminal and glandular epithelia of ovariectomised rats receiving E treatment. A relatively lower distribution of this protein was observed in the luminal epithelium of ovariectomised rats which received quercetin treatment.

**Fig 4:**
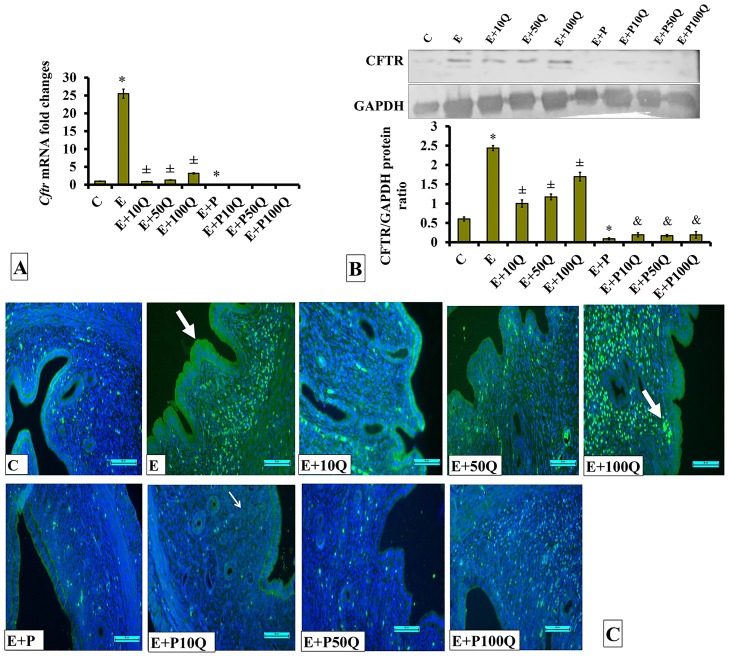
(A) *Cftr* mRNA levels, (B) Representative Western blot image and ratio of CFTR /GAPDH and (C) CFTR distribution in the endometrium of sex-steroid replaced ovariectomized rats with concomitant treatment with quercetin. Data were expressed as mean +/- SEM. N = 6 per treatment group. For A-C, *p < 0.05 compared to C, ±p<0.05 compared to E. ^&^ p<0.05 compared to E+P. Arrows show protein distribution.

In sex-steroid treated ovariectomised rats, treatment with E resulted in approximately 25-fold increase in *Cftr* mRNA level in uterus (Fig 4A). In E-treated ovariectomized rats, *Cftr* mRNA level markedly decreased following co-treatment with quercetin. Greatest decrease was observed following co-treatment with 10mg/kg/day quercetin. Meanwhile, in E+P-treated rats, low *Cftr* mRNA level was observed in the uterus, approximately 25-fold lower than E-treated rats. In these rats, no changes in *Cftr* mRNA level were observed following co-treatment with quercetin. CFTR protein level was highest in the uterus of E-treated ovariectomized rats (Fig 4B). In E-treated rats, the level of this protein markedly decreased following quercetin co-treatment. Greatest decrease was observed following co-treatment with 10mg/kg/day quercetin. Meanwhile, low CFTR protein level was observed in the uterus of E+P-treated rats, which was 2-fold lower than E-treated rats. In these rats, CFTR protein levels slightly increased following quercetin co-treatment (p<0.05). Immunofluorescence images show CFTR protein was highly distributed at apical membrane of endometrial luminal and glandular epithelia and in stroma of E-treated rats (Fig 4C). Relatively lower CFTR distribution was observed in E-treated rats receiving 10mg/kg/day quercetin treatment. In E-treated rats receiving co-treatment with 100mg/kg/day quercetin, relatively higher CFTR distribution was observed in the uterus compared to E-treated rats receiving 10 and 50mg/kg/day quercetin treatment. Low CFTR protein distribution was observed in the endometrium of E+P-treated rats with no obvious changes observed following quercetin co-treatment.

### Effects of quercetin-only and quercetin plus sex-steroids on SLC26A6 protein and mRNA expression and its protein distribution in the uterus

**Fig 5:**
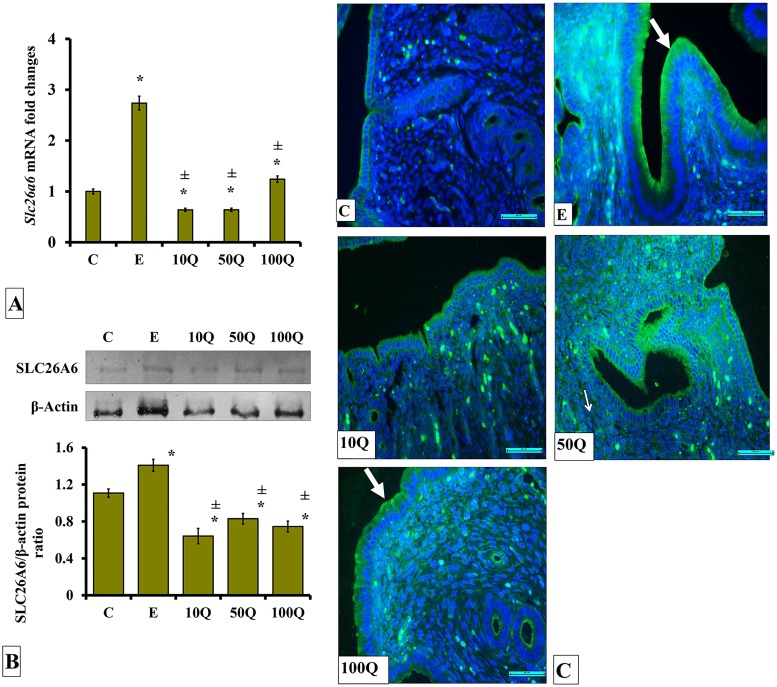
(A) *Slc26a6* mRNA levels, (B) Representative Western blot image and ratio of SLC26A6/GAPDH and (C) SLC26A6 distribution in the endometrium of ovariectomized, quercetin-treated rats. Data were expressed as mean +/- SEM. N = 6 per treatment group. For A-C, *p < 0.05 compared to C, ±p<0.05 compared to E. ^&^ p<0.05 compared to E+P.

*Slc26a6* mRNA level was highest in the uterus of ovariectomised, non-sex-steroid replaced rats receiving E treatment (Fig 5A). Levels of *Slc26a6* mRNA increased with increasing quercetin doses however did not surpass that following E treatment. SLC26A6 protein levels were highest in ovariectomised, non-sex-steroid replaced rats receiving E treatment (Fig 5B). In these rats, quercetin treatment caused low SLC26A6 protein expression level in uterus. Immunofluorescence images (Fig 5C) show SLC26A6 protein was highly distributed in endometrial luminal epithelium of rats receiving E treatment, mainly at the apical membrane SLC26A6 protein could also be seen to be distributed in luminal and glandular mainly following 50 and 100mg/kg/day quercetin treatment.

**Fig 6:**
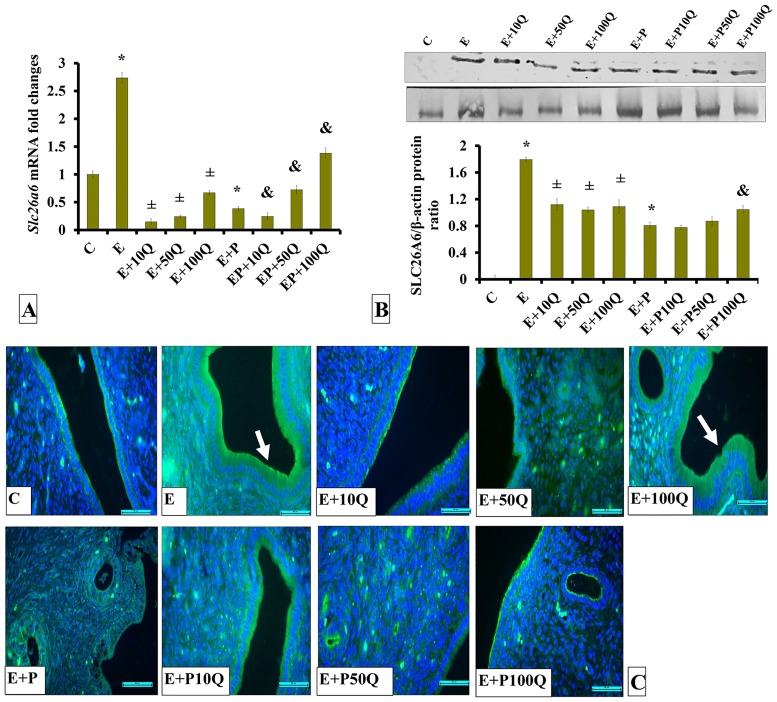
(A) *Slc26a6* mRNA levels, (B) Representative Western blot image and ratio of SLC26A6/GAPDH and (C) SLC26A6 distribution in the endometrium of sex-steroid replaced ovariectomized rats with concomitant treatment with quercetin. Data were expressed as mean +/- SEM. N = 6 per treatment group. *p < 0.05 compared to C, ±p<0.05 compared to E. ^&^ p<0.05 compared to E+P. Arrows show protein distribution.

In sex-steroid-replaced ovariectomised rats, levels of *Slc26a6* mRNA were highest following E-only treatment (approximately 2.6-folds higher than control) (Fig 6A). Co-administration of quercetin to E-treated rats resulted in *Slc26a6* mRNA levels in the uterus to significantly decreased (p<0.05). Greatest decrease was observed following co-administration of 10mg/kg/day quercetin. Meanwhile, in E+P-treated rats, levels of *Slc26a6* mRNA were lower than in E treated rats, approximately 5-fold. In these rats, co-treatment with 50 and 100mg/kg/day quercetin resulted in *Slc26a6* mRNA level in uterus to increase (p<0.05).

In ovariectomised rats, levels of SLC26A6 protein in uterus were highest following E treatment (Fig 6B). In E-treated rats, co-administration of quercetin resulted in SLC26A6 protein expression level to decrease (P<0.05). There were no significant differences in the level of this protein between quercetin doses. In E+P-treated rats, levels of SLC26A6 protein were markedly lower than in E-treated rats, by approximately 2-fold. In these rats, co-administration of 10 and 50mg/kg/day quercetin did not cause significant changes to SLC4A4 protein level but the level increased following 100mg/kg/day quercetin co-treatment (p<0.05). Immunofluorescence images show SLC626 protein was highly distributed at the apical membrane of endometrial luminal epithelium of E-treated rats (Fig 6C). In these rats, relatively lower SLC26A6 distribution was observed in the endometrium following quercetin co-treatment. A slightly higher SLC26A6 distribution was observed in the endometrium of E-treated rats receiving 100mg/kg/day quercetin treatment as compared to E-treated rats receiving 10mg/kg/day quercetin co-treatment. Very low amount of SLC26A6 protein was distributed in endometrium of E+P-treated rats. In these rats, a relatively higher distribution was observed following co-treatment with 100mg/kg/day quercetin.

### Effects of quercetin-only and quercetin plus sex-steroids on SLC4A4 protein and mRNA expression and its protein distribution in the uterus

**Fig 7:**
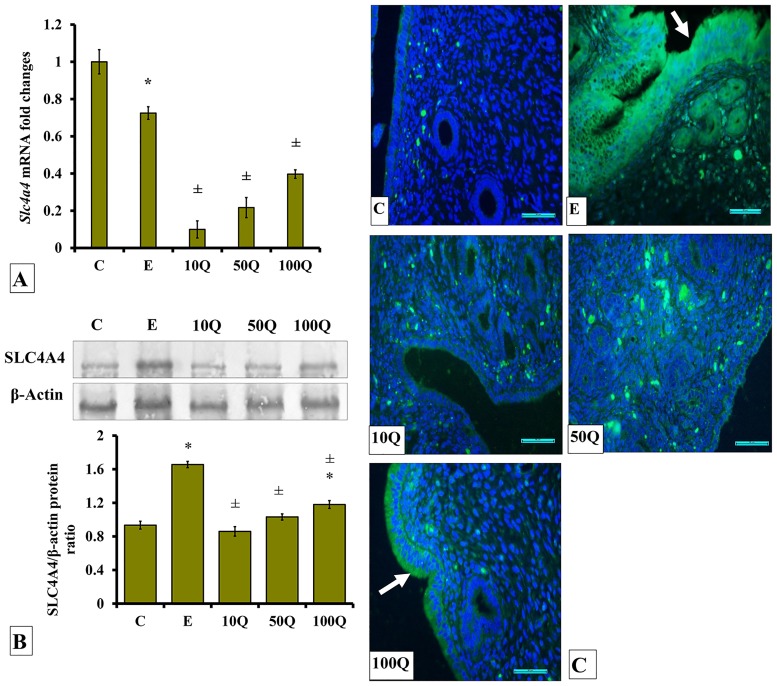
(A) *Slc4a4* mRNA levels, (B) Representative Western blot image and ratio of SLC4A4/β-actin and (C) SLC4A4 distribution in the endometrium of ovariectomized, quercetin-treated rats. Data were expressed as mean +/- SEM. N = 6 per treatment group. For A-C, *p < 0.05 compared to C, ±p<0.05 compared to E. ^&^ p<0.05 compared to E+P.

*Slc4a4* mRNA levels in the uterus of ovariectomized, non-sex-steroid-treated rats receiving E treatment were lower than control (Fig 7A). Treatment with quercetin resulted in a dose-dependent increase in *Slc4a4* mRNA levels, however did not surpass that in E treated rats. In ovariectomized, non-sex-steroid treated rats, the highest SLC4A4 protein levels were observed in the uterus following E treatment (Fig 7B). In quercetin-treated ovariectomised rats, a dose-dependent increase in SLC4A4 protein levels were observed in the uterus, however did not surpass the levels in E-treated rats. Immunofluorescence images (Fig 7C) show highest SLC4A4 protein distribution in the endometrium of E-treated ovariectomised rats. Relatively lower distribution was observed in the endometrium of rats receiving quercetin treatment. Treatment with 100mg/kg/day quercetin resulted in a relatively higher SLC4a4 distribution in the endometrium when compared to the rats receiving 10mg/kg/day quercetin treatment.

**Fig 8:**
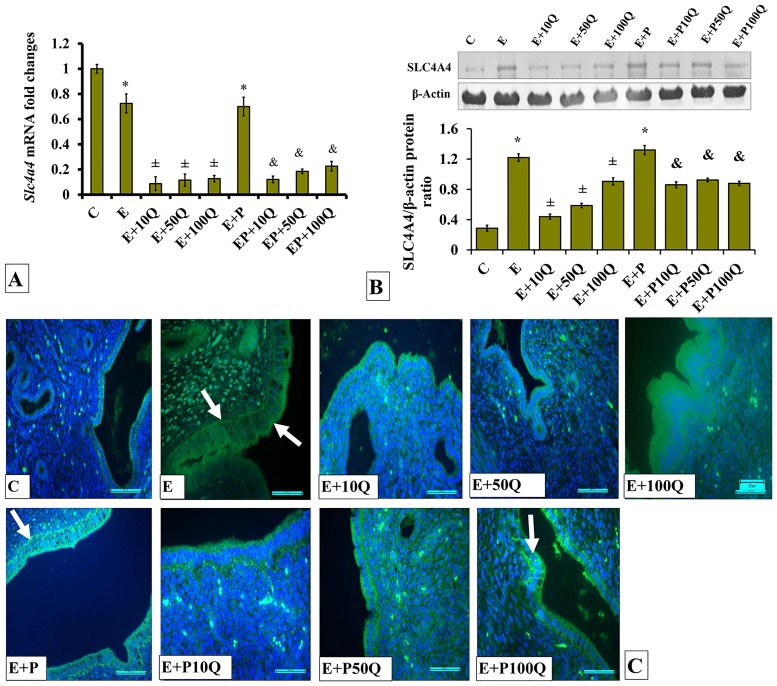
(A) *Slc4a4* mRNA levels, (B) Representative Western blot image and ratio of SLC4A4/GAPDH and (C) SLC4A4 distribution in the endometrium of sex-steroid replaced ovariectomized rats with concomitant treatment with quercetin. Data were expressed as mean +/- SEM. N = 6 per treatment group. *p < 0.05 compared to C, ±p<0.05 compared to E. ^&^ p<0.05 compared to E+P. Arrows show protein distribution.

*Slc4a4* mRNA levels in E-treated rats were markedly decreased following co-administration with quercetin (Fig 8A). No significant differences were observed in *Slc4a4* mRNA levels between different quercetin doses. In E+P-treated rats, *Slc4a4* mRNA levels were slight but significantly lower when compared to E-treated rats. In these rats, co-administration of quercetin resulted in *Slc4a4* mRNA levels in the uterus to markedly decrease.

The levels of SLC4A4 protein were highest in the uterus of ovariectomised rats receiving E treatment (Fig 8B). In these rats, co-administration of quercetin resulted in SLC4A4 protein levels to decrease. Greatest decrease was observed following co-administration of 10mg/kg/day quercetin with E. Meanwhile, in E+P-treated rats, the levels of SLC4A4 protein in the uterus were found to be approximately similar to the rats receiving E treatment. In these rats, co-administration of quercetin resulted in SLC4A4 protein levels in the uterus to significantly decrease.

Immunofluorescence images show high distribution of SLC4A4 protein in the endometrium of E-only treated ovariectomized rats (Fig 8C). This protein could be seen to be distributed in the luminal and glandular epithelia and stroma. Very low distribution was observed in E-treated rats receiving 10mg/kg/day quercetin treatment. However, co-administration of E with 100mg/kg/day quercetin resulted in a relatively higher SLC4A4 protein distribution in the luminal epithelium when compared to E-treated rats co-administered with 10mg/kg/day quercetin.

### Effects of quercetin-only and quercetin plus sex-steroids on α-ENaC protein and mRNA expression and its protein distribution in the uterus

**Fig 9:**
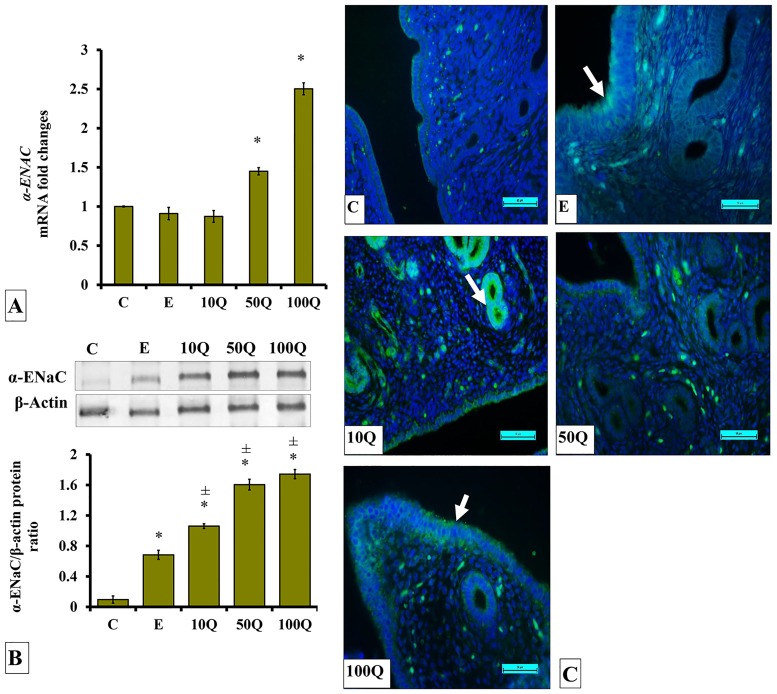
(A) *α-Enac* mRNA levels, (B) Representative Western blot image and ratio of α-ENaC /β-actin and (C) α-ENaC distribution in the endometrium of ovariectomized, quercetin-treated rats. Data were expressed as mean +/- SEM. N = 6 per treatment group. For A-C, *p < 0.05 compared to C, ±p<0.05 compared to E. ^&^ p<0.05 compared to E+P.

*α-Enac* mRNA levels in the uterus of ovariectomized, non-sex-steroid replaced rats were highest following 100mg/kg/day quercetin treatment (Fig 9A). A slight but significantly higher *α-Enac* mRNA levels were observed in the uterus of E-treated rats as compared to control. In ovariectomized rats which received 50 and 100mg/kg/day quercetin treatment, *α-Enac* mRNA levels in the uterus were not significantly different from control, however the levels were markedly increased following treatment with 100mg/kg/day quercetin.

In non-sex-steroid-treated ovariectomized rats, α-ENaC protein levels were highest following 50 and 100 mg/kg/day quercetin treatment (Fig 9B). Lower expression level of this protein was observed in E-treated ovariectomised rats and in rats receiving 10mg/kg/day quercetin treatment. Immunofluorescence images show α-ENaC protein was distributed in the luminal and glandular epithelia mainly in quercetin-treated ovariectomized rats (Fig 9C). Low distribution was seen in E-treated ovariectomised rats. This protein could be seen to be localized at the apical and basolateral membranes.

**Fig 10:**
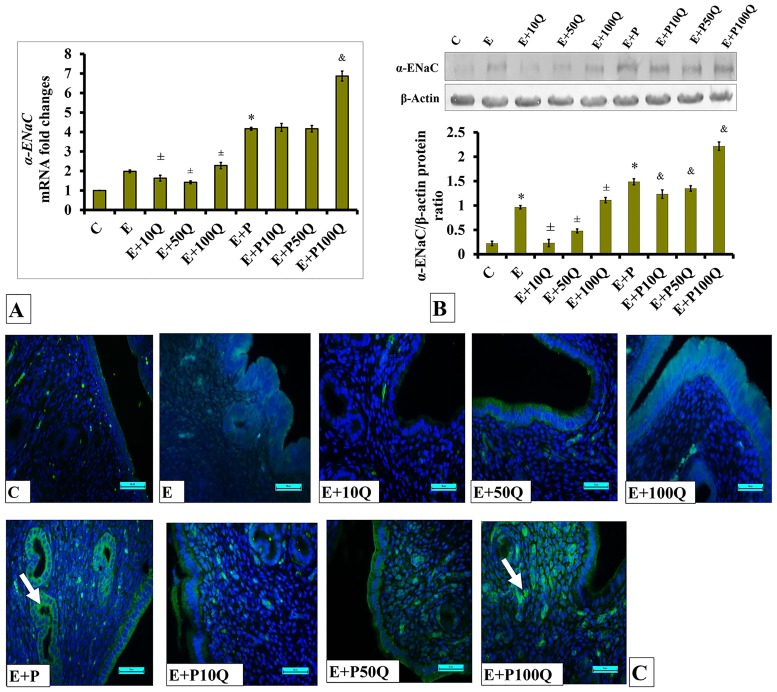
(A) *α-Enac* mRNA levels, (B) Representative Western blot image and ratio of α-ENaC /GAPDH and (C) α-ENaC distribution in the endometrium of sex-steroid replaced ovariectomized rats with concomitant treatment with quercetin. Data were expressed as mean +/- SEM. N = 6 per treatment group. *p < 0.05 compared to C, ±p<0.05 compared to E. ^&^ p<0.05 compared to E+P. Arrows show protein distribution.

In sex-steroid-replaced ovariectomized rats, concomitant administration of quercetin at 10mg/kg/day with E did not cause significant changes to *α-ENaC* mRNA level in the uterus (Fig 10A). In E-treated rats, significantly higher *α-ENaC* mRNA level was observed in the uterus following co-treatment with 50 and 100mg/kg/day quercetin. Meanwhile, in E+P-treated rats, higher *α-ENaC* mRNA levels were observed when compared to E-treated rats by approximately 2-folds. In these rats, significant increase in *α-ENaC* mRNA levels was observed following co-administration of 100mg/kg/day quercetin however not following co-administration with 10 and 50mg/kg/day quercetin.

Levels of α-ENaC protein in E-treated ovariectomized rats were markedly decreased following co-treatment with 10mg/kg/day quercetin (Fig 10B). Lesser decrease was observed following co-treatment of 50mg/kg/day quercetin. However, co-treatment with 100mg/kg/day quercetin caused α-ENaC protein level in the uterus to be slightly higher as compared to rats receiving E-only treatment. Meanwhile, in E+P-treated ovariectomized rats, the levels of α-ENaC protein in the uterus were found to be significantly higher as compared to E-treated ovariectomized rats (p<0.05). In these rats, slight but significant decrease in α-ENaC protein levels were observed in the uterus following co-administration of 10 and 50mg/kg/day quercetin. However, the level of this protein significantly increased following co-administration of 100mg/kg/day quercetin with E+P.

Immunofluorescence images (Fig 10C) show low distribution of α-ENaC protein in the endometrium of E-replaced ovariectomized rats. In these rats, distribution of α-ENaC protein in the endometrium was relatively higher following co-treatment with 50 and 100mg/kg/day quercetin. This protein could be seen to be distributed in the endometrial luminal epithelium both at the apical and basolateral membranes. A relatively higher α-ENaC protein could be seen to be distributed in the luminal and glandular epithelia of E+P-treated ovariectomized rats as compared to E-treated ovariectomized rats. This protein could be seen to be distributed at the apical and basolateral membranes. Co-administration of 100mg/kg/day quercetin to E+P-treated rats resulted in a relative increase in α-ENaC protein distribution in the luminal endometrium.

### Effects of quercetin-only and quercetin plus sex-steroids on β-ENaC protein and mRNA expression and its protein distribution in the uterus

**Fig 11:**
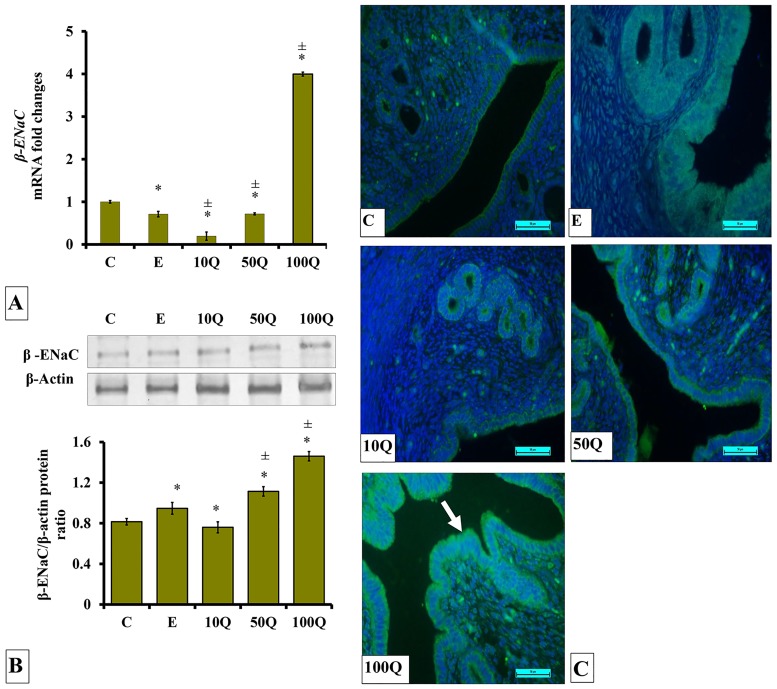
(A) *β-Enac* mRNA levels, (B) Representative Western blot image and ratio of β-ENaC /β-actin and (C) β-ENaC distribution in the endometrium of ovariectomized, quercetin-treated rats. Data were expressed as mean +/- SEM. N = 6 per treatment group. For A-C, *p < 0.05 compared to C, ±p<0.05 compared to E. ^&^ p<0.05 compared to E+P.

In ovariectomised, non-steroid replaced rats, the highest *β-ENaC* mRNA levels were observed following treatment with 100mg/kg/day quercetin, approximately four-fold higher as compared to control (Fig 11A). Very low *β-ENaC* mRNA level was observed in the uterus of E-treated ovariectomized rats. Treatment with 10 and 50mg/kg/day quercetin resulted in *β-ENaC* mRNA levels in the uterus to be slight but significantly higher than in E-treated rats.

Highest β-ENaC protein level could be seen in the uterus of ovariectomized rats receiving 100mg/kg/day quercetin treatment (Fig 11B). A slightly higher β-ENaC protein level was observed in E-treated rats than control rats but was approximately 1½ fold lower than in rats receiving 100mg/kg/day quercetin treatment. A slight but significantly higher β-ENaC protein level could also be seen in rats receiving 50mg/kg/day quercetin treatment as compared to the rats receiving E-only treatment. Immunofluorescence images show low distribution of β-ENaC protein in the endometrium of E-treated ovariectomized rats (Fig 11C). A relatively higher β-ENaC protein distribution was observed in the uterus following quercetin treatment as compared to E treatment. This protein was found to be distributed in the luminal and glandular epithelia mainly at the apical and basolateral membranes.

**Fig 12:**
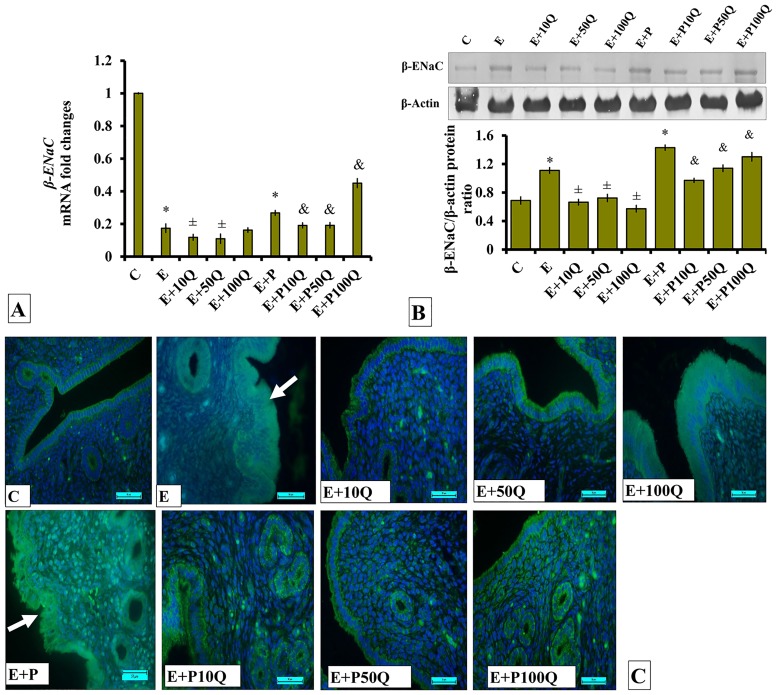
(A) *β-Enac* mRNA levels, (B) Representative Western blot image and ratio of β-ENaC /GAPDH and (C) β-ENaC distribution in the endometrium of sex-steroid replaced ovariectomized rats with concomitant treatment with quercetin. Data were expressed as mean +/- SEM. N = 6 per treatment group. *p < 0.05 compared to C, ±p<0.05 compared to E. ^&^ p<0.05 compared to E+P. Arrows show protein distribution.

Low *β-ENaC* mRNA levels were observed in the uterus of E-treated ovariectomized rats (Fig 12A). In these rats, *β-ENaC* mRNA levels slightly decreased following co-treatment with 10 and 50mg/kg/day quercetin. Levels of *β-ENaC* mRNA in E+P-treated rats were significantly higher than E-treated rats (P<0.05). In these rats, a slight but significant decrease in *β-ENaC* mRNA levels was observed in the uterus following 10 and 50mg/kg/day quercetin co-treatments, however was significantly increased following 100mg/kg/day quercetin treatment (p<0.05).

β-ENaC protein levels in the uterus of ovariectomized rats receiving E treatment markedly decreased following co-administration of quercetin at all three doses (p<0.05) (Fig 12B). Higher β-ENaC protein level was observed in the uterus of E+P-treated rats as compared the rats receiving E-only treatment. In these rats, co-administration of quercetin resulted in β-ENaC protein level in the uterus to decrease. Lesser decrease was observed following co-treatment with 100mg/kg/day quercetin as compared to co-treatment with 10mg/kg/day quercetin.

Immunofluorescence images (Fig 12C) show highest distribution of β-ENaC protein in the endometrium of E+P-treated rats. In these rats, β-ENaC protein could be seen to be distributed in the luminal and glandular epithelia. Co-administration of 10 and 50mg/kg/day quercetin with E+P resulted in a relatively lower β-ENaC protein distribution in the endometrium. Low distribution of β-ENaC protein could be seen in E-treated ovariectomized rats’ endometrium. In these rats, a relatively higher β-ENaC protein could be seen to be distributed in the luminal epithelium following co-treatment with 50 and 100mg/kg/day quercetin.

### Effects of quercetin-only and quercetin plus sex-steroids on γ-ENaC protein and mRNA expression and its protein distribution in the uterus

**Fig 13:**
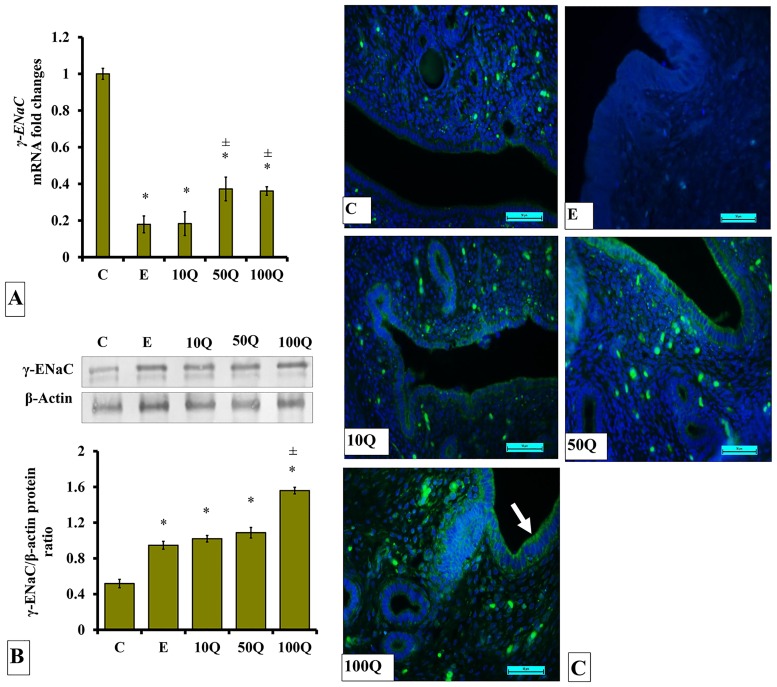
(A) *γ-Enac* mRNA levels, (B) Representative Western blot image and ratio of γ-ENaC /β-actin and (C) γ-ENaC distribution in the endometrium of ovariectomized, quercetin-treated rats. Data were expressed as mean +/- SEM. N = 6 per treatment group. For A-C, *p < 0.05 compared to C, ±p<0.05 compared to E. ^&^ p<0.05 compared to E+P.

In E-treated ovariectomized rats, low levels of *γ-Enac* mRNA were expressed in the uterus (Fig 13A). Treatment with 50 and 100mg/kg/day quercetin resulted in higher *γ-Enac* mRNA levels as compared to E treatment.

Levels of γ-ENaC protein were highest in ovariectomized rats which received 100mg/kg/day quercetin treatment (Fig 13B). In E-treated ovariectomized rats, γ-ENAC protein level in uterus were lower than in rats receiving 100mg/kg/day quercetin treatment, by approximately 2-folds however were not significantly different when compared to the rats receiving treatment with 10 and 50mg/kg/day quercetin. Immunofluorescence images (Fig 13C) show low distribution of γ-ENAC protein in the endometrium of ovariectomized rats receiving E treatment. Relatively higher γ-ENaC protein was distributed in the endometrium of rats receiving 100mg/kg/day quercetin as compared to the rats receiving E treatment, mainly in the stroma and luminal epithelium.

**Fig 14:**
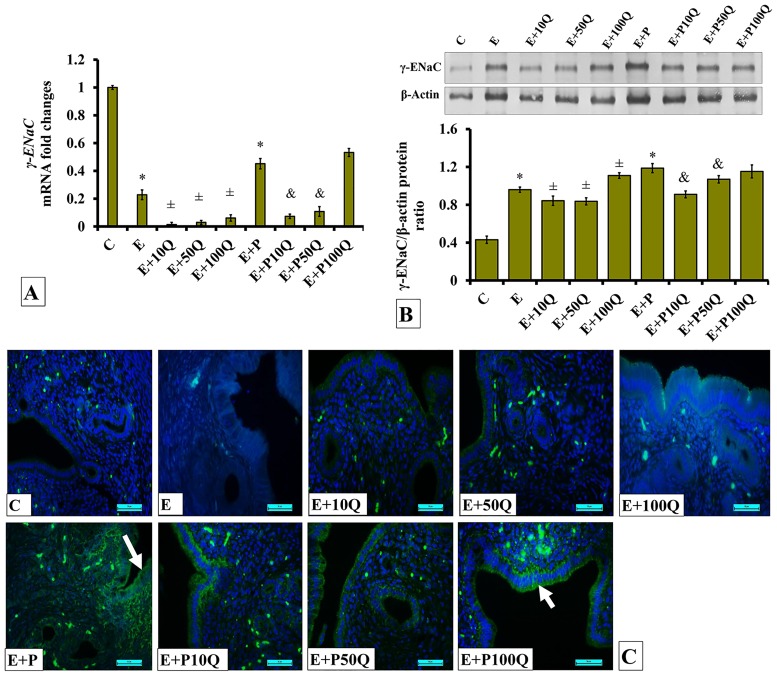
(A) *γ-Enac* mRNA levels, (B) Representative Western blot image and ratio of γ-ENaC /GAPDH and (C) γ-ENaC distribution in the endometrium of sex-steroid replaced ovariectomized rats with concomitant treatment with quercetin. Data were expressed as mean +/- SEM. N = 6 per treatment group. *p < 0.05 compared to C, ±p<0.05 compared to E. ^&^ p<0.05 compared to E+P. Arrows show protein distribution.

In E-treated ovariectomized rats, levels of *γ-Enac* mRNA were markedly reduced following 10mg/kg/day quercetin treatment (Fig 14A). Lesser reduction was observed following co-treatment with 50 and 100mg/kg/day quercetin. Higher *γ-Enac* mRNA level could be seen in ovariectomized rats receiving E+P treatment as compared to E treatment. In these rats, co-administration of 10 and 50mg/kg/day quercetin resulted in markedly decreased in *γ-Enac* mRNA level, however, no changes was observed in the level of *γ-Enac* mRNA following co-treatment with 100mg/kg/day quercetin.

The level of γ-ENaC protein in E-treated ovariectomized rats was reduced following concomitant treatment with 10 and 50mg/kg/day quercetin (Fig 14B). However, co-treatment with 100mg/kg/day quercetin resulted in γ-ENaC protein level in the uterus to slightly increase. Higher γ-ENaC protein level was observed in the uterus of E+P-treated rats as compared to E-treated rats (p<0.05). In these rats, levels of γ-ENaC protein markedly decreased following co-treatment with 10 and 50 mg/kg/day quercetin.

Immunofluorescence images (Fig 14C) show low distribution of γ-ENaC protein in the endometrium of ovariectomized rats receiving E treatment. In these rats, no differences in γ-ENaC protein distribution was observed in the endometrium following co-administration with quercetin. Higher γ-ENaC protein distribution was observed in the endometrium of E+P-treated ovariectomized rats as compared to E-treated ovariectomized rats. In the former, quercetin co-treatment did not cause marked changes to the γ-ENaC protein distribution in the uterus. γ-ENaC protein could be seen to be localized at the apical and basolateral membranes of the luminal and glandular epithelia.

### Effects of quercetin-only and quercetin plus sex-steroids on expression and distribution of Na^+^-K^+^-ATPase protein in the uterus

**Fig 15:**
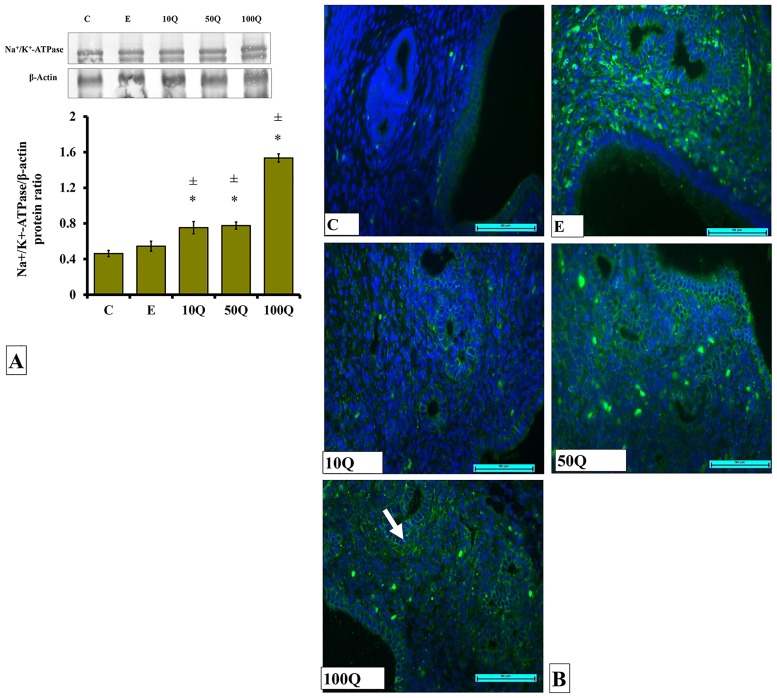
(A) Representative Western blot image and ratio of Na^+^/K^+^-ATPase/β-actin and (B) Na^+^/K^+^-ATPase distribution in the endometrium of ovariectomized, quercetin-treated rats. Data were expressed as mean +/- SEM. N = 6 per treatment group. For A-C, *p < 0.05 compared to C, ±p<0.05 compared to E. ^&^ p<0.05 compared to E+P. Arrows show protein distribution.

High expression of Na^+^-K^+^-ATPase protein could be seen in ovariectomized rats receiving 100mg/kg/day quercetin treatment (Fig 15A). In ovariectomized rats receiving E treatment, low expression of this protein could be seen in the uterus, approximately 3 fold lower than in rats receiving 100mg/kg/day quercetin treatment. Immunofluorescence images show Na^+^-K^+^-ATPase protein was distributed at the basolateral membrane with high distribution in rats receiving 50 and 100mg/kg/day quercetin treatment (Fig 15B).

**Fig 16:**
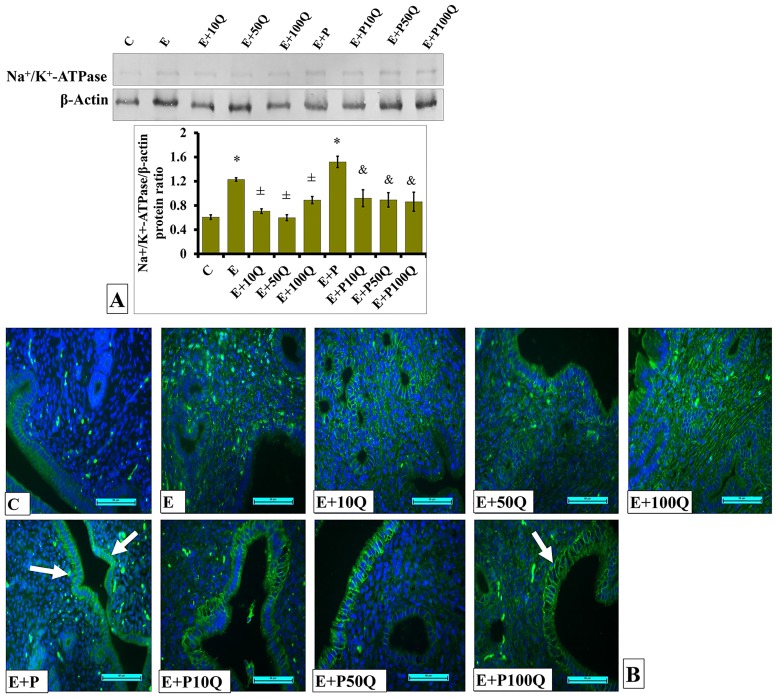
(A) Representative Western blot image and ratio of Na^+^/K^+^-ATPase/GAPDH. (B) Na^+^/K^+^-ATPase distribution in the endometrium of sex-steroid replaced ovariectomized rats with concomitant treatment with quercetin. Data were expressed as mean +/- SEM. N = 4 per treatment group. *p < 0.05 compared to C, ±p<0.05 compared to E. ^&^ p<0.0*5* compared **to** E+P. Arrows show protein distribution.

In ovariectomized rats receiving E treatment, expression level of Na^+^-K^+^-ATPase protein was markedly decreased following co-administration of quercetin at all three doses (Fig 16A). Levels of Na^+^-K^+^-ATPase protein in the uterus was higher in E+P-treated ovariectomized rats when compared to E-treated ovariectomized rats. In these rats, quercetin co-treatment resulted in the levels of Na^+^-K^+^-ATPase protein in the uterus to decrease. Immunofluorescence images show low distribution of Na^+^-K^+^-ATPase protein in ovariectomized rats which received E treatment (Fig 16B). In these rats, co-administration of quercetin caused no remarkable changes in distribution of Na^+^-K^+^-ATPase protein in the endometrium. High distribution of Na^+^-K^+^-ATPase protein could be seen in the luminal and glandular epithelia of E+P-treated ovariectomized rats, mainly at the basolateral membrane. Co-adminsitration of quercetin did not cause marked changes in Na^+^-K^+^-ATPase protein distribution in the endometrium.

## Discussion

This study has revealed for the interference of sex-steroid regulation of uterine fluid volume and its electrolytes’ concentrations by quercetin. Administration of quercetin in the absence of sex-steroids was able to cause increased in fluid secretion rate as well as HCO_3_^-^ concentration in uterine fluid despite of no changes on uterine fluid Na^+^ and Cl^-^ concentrations. In view that E causes increased in uterine fluid secretion rate and HCO_3_^-^ concentration, therefore there was a possibility that high dose quercetin could displays effects resembling E. E-like quercetin effects have been reported elsewhere [17].

However, in the presence of sex-steroids, quercetin displayed different effects i.e. it antagonizes E effect on all the measured parameters. Low dose quercetin (10mg/kg/day) exerted the greatest inhibition in the presence of E which indicated that at low dose, quercetin could possibly displayed anti-estrogenic activity. Similar pattern of quercetin’s effects were reported by Del Medina et al, [26] who observed that at low dose i.e. 10 mg/kg/day, quercetin exerted greater inhibition on the epithelial Cl^-^ secretion as compared to at high dose i.e. 100 mg/kg/day. These peculiar effects might be due quercetin’s ability to modulate E receptors (ERs) and DNAs. Being a compound with weak estrogenic activities, at low dose quercetin might inhibit estrogen actions. However, at high dose, quercetin could directly stimulates estrogen-response elements (ERE) on the DNAs, which would produce lesser inhibition. There is a possibility that high dose quercetin possesses anti-progestogenic effects as it was found to antagonize P effects on Na^+^ and HCO_3_^-^ concentrations in rats receiving E+P treatment.

The mechanisms that could explain changes to the above parameters have also been explored. Quercetin could have exerted its’ effect through the GP-ACII-CAMP signaling pathways that is known to participate in ion channels/membrane transporters’ functions [27, 28] ACII-GP-cAMP has been shown to be involved in CFTR and SLC26A6 activation which could lead to the increased in HCO_3_^-^ secretion. [29]. cAMP is required for SLC26A6 and CFTR functions in the pancreas and salivary ductal epithelial cell [30]. GP-ACII-cAMP pathway has also been found to play important role in enhancing Na^+^ transport in the epithelia via activation of ENaC and Na^+^/K^+^-ATPase functions in the lungs which stimulates the airway fluid clearance [31]. Reduced level GP-ACII-cAMP in uterus under E influence following low dose quercetin i.e. 10mg/kg/day treatment could disturbed the regulation of uterine fluid electrolytes by E. Low uterine secretory activities under P influence could be due to low expression of uterine GPα, GPβ and ACII and low level of cAMP, causing inactivation of the membrane transporters’ function. Therefore the increased in Na^+^ and HCO_3_^-^ secretions in E+P-treated rats receiving high dose quercetin treatment could be due to other mechanisms rather than the activation of GP-ACII-CAMP pathways.

In this study, quercetin was found unable to stimulate expression of uterine CFTR. When low dose quercetin was co-administered with E, greatest inhibition of CFTR expression was observed which further strengthened the views that at low dose, quercetin exerts anti-estrogenic effects. Low CFTR expression in the uterine luminal and glandular epithelia following co-treatment with quercetin, could reduce endometrial Cl^-^ and HCO_3_^-^ secretions, which results in reduction of the Cl^-^ and HCO_3_^-^ concentrations of the uterine fluid. Meanwhile, the lack of effect of quercetin on CFTR expression level in rats receiving E+P-treatment indicated that quercetin was not able to interfere with P effects in the uterus which could be due to the possibility that quercetin was not able to antagonize P actions. Although there was no changes in the Cl^-^ concentration following co-administration of quercetin with E+P, but marked increase in HCO_3_^-^ concentration particularly when high dose quercetin was concomitantly administered was observed. These indicated that other HCO_3_^-^ transporter apart from CFTR could be involved in enhancing the uterine fluid HCO_3_^-^ concentrations.

The ability of high dose quercetin to stimulate SLC26A6 expression in the uterus could account for the increased in uterine fluid HCO_3_^-^ concentration. In the presence of E, quercetin was able to suppress *Slc26a6* mRNA and protein expressions which likely to cause decreased in the uterine fluid HCO_3_^-^ concentration as observed under this condition. Meanwhile, increased in SLC26A6 expression in E+P-treated rats receiving high dose quercetin treatment could account for the increased in uterine fluid HCO_3_^-^ concentration as observed under this condition.

In the meantime, increased in uterine fluid HCO_3_^-^ following increasing dose quercetin treatment could also be due to increase in SLC4A4 expression, which participates in the transport of Na^+^ and HCO_3_^-^ into the uterine lumen. It was found that under E influence, where SLC4A4 expression in the uterus was known to be up-regulated [10], administration of quercetin markedly reduced the *Slc4a4* mRNA and protein expressions which could account for the observed reduction in uterine fluid Na^+^ and HCO_3_^-^ concentrations as observed under this condition. Meanwhile, under E+P influence, reduced expression of SLC4A4 following quercetin co-treatment could possibly account for the increased in Na^+^ and HCO_3_^-^ concentrations of the uterine fluid as observed under this condition. This is likely due to reduce expression of basolaterally-located SLC4A4 that participates in Na^+^ and HCO_3_^-^ extrusion from the cells.

Quercetin was found able to affect the Na^+^ concentration of uterine fluid, most probably via affecting expression of Na^+^ channels in the endometrium. High dose quercetin was found able to stimulate expression of α, β and γ-ENaC proteins in the uterus, which could result in reduced concentration of uterine fluid Na^+^. The observed low expression of ENaC, a channel known to participate in luminal Na^+^ reabsorption, under E influence could help to maintain the high Na^+^ levels in the uterine fluid, which is secreted into the lumen through the apically-located Na^+^/HCO_3_^-^ co-transporter (SLC4A4) in the endometrial epithelium.

Expression of α, β and γ-ENaC in the uterus was found highest in rats receiving E+P treatment, consistent with other findings [11]. Under this condition α, β and γ-ENaC expressions in the uterus markedly decreased following co-administration of 10 and 50mg/kg/day quercetin. These could lead to decreased Na^+^ reabsorption from the uterine lumen, subsequently maintaining high levels of Na^+^ in the uterine fluid. Despite of a remarkably increased α-ENaC expression following high dose (100mg/kg/day) quercetin treatment in rats receiving E+P, no further decrease in uterine fluid Na^+^ was observed. These could be due to the presence of non-functioning ENaC as only expression of α but not β or γ subunits’ were increased under this condition. In the meantime, under E influence, expression of ENaC subunits were also reduced following co-administration of low dose quercetin which could help to sustain high Na^+^ concentration in the uterine fluid. However Na^+^ concentration was found to decreased, likely due to decrease expression of SLC4A4.

Administration of high dose quercetin was found able to up-regulate expression of Na^+^/K^+^-ATPase to the levels higher than that under E influence. These could result in lower uterine fluid Na^+^ concentration in view that Na^+^/K^+^-ATPase facilitates the removal of Na^+^ from the uterine lumen [11]. The marked reduction of Na^+^/K^+^-ATPase expression in the uterus following co-administration of quercetin with E would likely decreased Na^+^ reabsorption, therefore favors net Na^+^ secretion into the lumen. In contrast, under E+P influence, Na^+^/K^+^-ATPase expression in the uterus was high, the findings which were consistent with others [11, 32]. This would result in enhanced Na^+^ reabsorption which decreased Na^+^ level in the uterine fluid. Following co-administration of quercetin, Na^+^/K^+^-ATPase expression were markedly decreased, where this could contribute towards increased in uterine fluid Na^+^ levels via decreasing the luminal Na^+^ reabsorption.

In conclusions, quercetin was found able to affect uterine fluid secretion rate and Na^+^, Cl^-^ and HCO_3_ concentrations in the uterus via modulating expression of membrane transporters, channels and pump in the endometrial epithelia. The observed quercetin effects could potentially disturbed the delicate regulation of uterine fluid volume and its electrolytes concentration by sex-steroid hormones, in which these might have major implications on the uterine reproductive functions.
